# Young People and Intimate Partner Violence: Experiences of Institutional Support and Services in England

**DOI:** 10.1007/s10896-023-00591-x

**Published:** 2023-06-13

**Authors:** Maria Barnes, Christine Barter, Annie Herbert, Jon Heron, Gene Feder, Eszter Szilassy

**Affiliations:** 1 Department of Population Health Sciences, University of Bristol, Canynge Hall, Whatley Road, Bristol, BS8 2PS UK; 2 University of Central Lancashire, Preston, UK

**Keywords:** Intimate Partner Violence and Abuse, Young People, Services, Experience

## Abstract

**Purpose:**

Young people (YP) are at greater risk of experiencing intimate partner violence and abuse (IPVA), with high prevalence rates at 18–25 years and potentially severe short and long-term health and social impacts. YP often view adult support services as not for them and more research is needed to understand effective responses to IPVA among different groups.

**Methods:**

Semi-structured interviews alongside Life History Calendars were undertaken to explore 18 young peoples’ (18–25 years) experiences of community and service level responses to their IPVA between 2019–2020. Thematic analysis and case studies were carried out.

**Results:**

Participant accounts commonly described what did or did not help within: education; primary care physicians and maternity services; third sector or non-government support organisations; and counselling and support workers. YP wanted clearer information on identifying abuse from a younger age in schools and better access and signposting to specialist services. They benefited the most from equal power dynamics in relationships with professionals where they were supported to make their own decisions.

**Conclusions:**

Professionals in all sectors (including schools) need IPVA trauma-informed training that encourages equal power dynamics, with a clear understanding of and access to referral pathways, to be able to respond to the needs of YP experiencing IPVA.

## Background

Young people (YP) (18–25) are at particular risk of intimate partner violence and abuse (IPVA), defined as the physical, emotional/psychological, or sexual abuse by a current or former partner. IPVA sits within ‘domestic violence and abuse’ (DVA, which also includes violence and abuse between family members over 16) (Home Office, 2021). National UK crime surveys consistently report young women to be at greater risk of victimisation than those over 25 (ONS, 2021). Rates in adolescents and YP are high – with reported prevalence of up to 97% for emotional and psychological violence in a synthesis of current evidence on the prevalence and determinants of IPVA, where most of these studies were conducted in developed countries (Hossain et al., 2020). IPVA is one of the leading risks of death globally for younger women (20–24) and between 1990 to 2013 the burden of IPVA rose (Mokdad et al., 2016).

Although estimating prevalence of victimisation for young people is challenging, it is estimated that approximately 20% of young people experience physical victimisation from an intimate partner (Wincentak et al., 2017). A UK study found that around half of all young people experience emotional victimisation, between 50–70% experience intimate partner violence and abuse through technologies, and a quarter of adolescents report some type of unwanted sexual contact (Stonard et al., 2014).

In the latest figures from the Office of National Statistics for England and Wales (2022), the prevalence of IPVA is highest in the 20–24 years group (6.3%) as compared to the age 35–44 group with the next highest prevalence (4.2%).

Øverlien et al. (2020) states that there are, arguably, aspects of IPVA that are different in YP compared to adults. Being young is a time of firsts in terms of relationships and sex, influencing expectations around what is normal behaviour. This can obscure the abuse for YP and make them less likely to discuss, say, sexual coercion and controlling behaviour with others. Research has also shown that YP’s pervasive use of digital media e.g. sharing passwords, monitoring behaviours, believing certain things about sexual intimacy, sharing intimate pictures – can put them at higher risk of IPVA (Baker & Carreño, 2016).

Few studies have focussed on the experiences of young women experiencing IPVA. Of those, (from Western, developed countries), it is reported that gendered: power relations, behavioural expectations, inequalities and social and cultural pressures can put young women at risk of experiencing IPVA in their intimate relationships, and remaining in them (Aghtaie et al., 2018; Chung, 2007; Ismail et al., 2007; Korkmaz & Øverlien, 2019; Toscano, 2014).

Meta-analysis by Park and Kim (2018) of YP between 13–22, showed that young women reported greater sexual violence victimisation compared to young men. In 13 -17 year olds in Europe (Barter et al., 2017) statistically significant differences were found in respect of gender and IPVA subjective impact: young women were more likely than young men to attribute a negative impact (feeling scared and upset) to their experiences of victimisation whilst young men were more likely to state an affirmative only impact (thought it was funny) or report no effect. Similarly, researchers in New Zealand (Jackson et al., 2000) found that girls reported more negative emotional responses to their experiences of IPVA than boys.

Though there is little evidence on outcomes following IPVA in YP, evidence on adolescents suggests that IPVA in youth is associated with a range of health impacts and risk behaviours including substance use and misuse, mental health problems, in particular post-traumatic stress, self-harm, suicidality and disordered eating (Authors’ own, 2020; Clutterbuck, 2019; Cole, 2019; McGregor, 2018; Barter & Stanley, 2016). The physical impacts of violent relationships can include sexually transmitted illness, miscarriages and hospitalisations (Cole, 2019; McGregor, 2018).

Qualitative evidence on IPVA and YP is thin (Fernet et al., 2019; Aghtaie et al., 2018; Gadd et al., 2015; Fox et al., 2014; Barter, 2009; Chung, 2007) primarily focussing on younger adolescents and little research examining YP interactions with services (Wood et al., 2011; Moore et al., 2015). The evidence-base indicates that adolescents suffering IPVA are most likely to disclose experiences to their peers (Aghtaie et al., 2018; Dragiewicz et al., 2018) although they do not necessarily get the support they need due to peers minimising, denying, or not knowing how to help, discouraging the IPVA survivor from disclosing again (Barter & Stanley, 2016). Many adolescents report feeling that teachers are not conducive to discussions about intimate relationships (Dragiewicz et al., 2018; Leen et al., 2013). Those who seek medical care for injuries often feel uncomfortable disclosing IPVA (Heron et al., 2022). Some minority groups, for example, LGBTQ + YP may have a particular mistrust of medical professionals, often fearing that they may be outed to their families (Hester & Donovan, 2014). Young mothers often express resentment towards social services, fearing the removal of their children due to the consequences of their abusive partner’s behaviour (Wood & Barter, 2015; Young et al., 2018).

Adolescents find formal support helpful when it is tailored to their needs and where they could speak with someone their own age who understood what they were going through (Young et al., 2018; SafeLives, 2017). Support is viewed as beneficial when, instead of attempting to force a young person to end the relationship, they are provided with information about unhealthy behaviours using accessible language and enabling them to make decisions for themselves. (SafeLives, 2017; SafeLives Report, 2020).

Despite high IPVA prevalence rates, YP remain a ‘hidden’ group when it comes to accessing services and support (SafeLives Report, 2021). Frontline community organisations highlight that this age group can ‘disappear through the gaps’ due to child and adolescent services stopping at the age of 18 with adult services being perceived as ‘not for them’ (Macnab, 2010). More qualitative research is needed to understand what is required from services to provide effective support for YP in abusive relationships.

The main aim of the original research was to explore the intergenerational transmission of domestic violence i.e. to investigate the mechanisms by which domestic violence and abuse (DVA) in the family of origin (that is, exposure to IPVA occurring between de-facto parents/caregivers whilst growing up) may or may not manifest in the intimate relationships of young people. Consequently, participants were recruited who had experienced DVA **and/or** IPVA. In this paper, however, we report findings on experiences of formal support from participants who had all experienced IPVA—most of whom had experienced both DVA and IPVA—and had sought or received institutional help and support primarily in relation to experiences of IPVA. The paper focuses on YPs experiences of institutional support, services, or professional groups for IPVA; what helped and what would help in terms of prevention, intervention, and support for YP in abusive relationships.

## Methods

As part of the larger mixed-methods study in England (YARAH Study, n.d.), qualitative, face-to-face semi-structured interviews using Life History Calendars (Freedman et al., 1988) were used to recall and explore respondents’ experiences in various domains (such as family and intimate relationships) from across their life-course.

Research on recall and survey methodologies has consistently found that the longer the reporting period between events and interview, the more likely it is for underreporting and inaccuracy by participants (Wagenaar, 1986; Yoshihama et al., 2005). The LHC method combines a visual calendar with a semi-structured interview schedule, helping participants better access to memories with the use of memory cues in recalling patterns of past events.

The interviewer explained the LHC at the start of the interview stating that it was a way to look back on life events and they could start anywhere and fill in the two A3 pieces of paper however they wanted (or the interviewer could).

The interview topic areas were developed collaboratively using the literature and multidisciplinary research team and Public Patient Involvement expertise. They included asking young people about: family; education; friendships; intimate relationships; mental and physical health; wellbeing and help-seeking. Within each area, probe questions were prepared that allowed for expansion of the topic.

Eligible participants were those between 18–25 years old who had experience of domestic violence in their family of origin **and/or** intimate partner violence and abuse in their own relationships. Ethical considerations guided recruitment to be only with young adults who had access to support—whether through frontline multi-agency organisations providing support/housing/advice, online support forums (such as for LGBT + community) or counselling services. Frontline service providers passed on information about the study to eligible participants where it was deemed safe to do so. These are not named within the paper to ensure confidentiality of the participants.

To ensure a range of participants were recruited information about the study was also cascaded through various LGBT + networks online, university media (targeted at those with access to university mental health support), and the YARAH study website.

In total 18 interviews (each with a different YP) were undertaken between August 2019 and August 2020, lasting between one hour and two hours 45 min. All were audio-taped and written consent was taken. Sixteen interviews were carried out face-to-face in participants’ homes, a private room in frontline organisation offices, or the university. In March 2020, due to the COVID-19 pandemic, four interviews were carried out via a confidential video platform and written consent was received from the participant. The researcher was careful to check on the safety of the participant prior to the interview (‘is it safe for you to discuss your experiences with me?’), emphasise the confidentiality and anonymity aspects of the study, as well checking that support and/or self-care mechanisms were in place after the interview. Case notes on were written up after each interview to capture timelines and any non-verbal cues.

Approval was received by University of Bristol Ethics Committee Approval (ref: 83722) in 2019 for the YARAH Study. All methods were carried out in accordance with relevant guidelines and regulations. Informed, written consent was obtained from all subjects.

## Analysis

The interview data were fully transcribed. Data collection and analysis occurred concurrently and iteratively and in case-studies according to the constant comparison methods of grounded theory (Glaser & Strauss, 1967). Thematic analysis (Braun & Clarke, 2012) was used to develop and refine clusters of themes from the data. Life History Calendar data were used as part of the participants’ case-studies to cross-check information and give a life-course narrative perspective.

Data relating to the first four interviews were analysed by detailed scrutiny of the transcripts, which were then coded with the aid of NViVo12 software. A coding comparison exercise then took place with other members of the research team (MB, ES, CAB) where codes were refined, and any discrepancies examined and discussed until consensus was reached. A framework was then developed and used to code further transcripts within the sample and across sets e.g. whether recruited through frontline agencies or through other avenues or whether participant had children or not.

Record keeping was meticulous, demonstrating a clear decision trail and ensuring interpretations of the data were consistent and transparent. Data were examined for similarities and differences within themes and within and across sets to ensure different perspectives were represented. Data saturation was reached in the themes presented i.e. when further sampling did not yield any new data. Case studies – in the form of the LHCs – were also compared. All names and identifying features have been changed.

A patient and public involvement engagement panel (PPIE) were recruited through the wider public engagement panel developed by PPIE Leads within the Centre for Academic Care within the University. Two young women with lived experience of DVA within their family of origin and IPVA in their own relationships were involved with guiding the current research, giving their perspectives to help develop: wording on the study adverts; on recruitment practices across the DVA frontline and other specialist organisations; adding to the topic guide; reflecting on data and possible themes and on how to disseminate study information beyond academia.

### Definitions

Domestic violence and abuse was defined as in the UK Domestic Abuse Act (Home Office, 2021) whereby behaviour is “abusive” if it consists of any of the following: physical or sexual abuse; violent or threatening behaviour; controlling or coercive behaviour; economic abuse; psychological, emotional or other abuse; and it does not matter whether the behaviour consists of a single incident or a course of conduct. IPVA is also domestic violence and abuse and consists of the same behaviours but refers specifically to abuse that occurs within an intimate relationship only, and not the wider family.

People and services will often use the term ‘domestic violence’ to cover both of definitions, which can sometimes blur the boundaries and make disentangling which particular experience people are describing difficult. However, this is how people experience and use the terminology and is, therefore, reflected in some of the findings.

## Results

Eighteen participants between 19 and 25 years old (16 women, one transgender male, one transgender female) were interviewed; seven of the women had children. Participants came from a range of socio-economic and ethnic backgrounds as shown in Tables 1, 2, 3.

**Table 1:** Characteristics of Young People (19–25 years)

DVA and IPVA	*n* = 18
Ethnicity	
- White British	13
- Black British/dual	2
- South Asian British/dual	2
- White Eastern Europe	1
Sexuality	
- Heterosexual	11
- Bisexual	2
- Pansexual	2
- Not know	3
Education (level achieved)	
- GCSE/NVQ/BTEC*	11
- A level**	2
- Degree started/completed	5
Child(ren)	**7**
DVA in Family of Origin	15
- Physical	10
- Sexual	5
- Emotional	9
- Control	5
- Neglect	8
Bullying by peers	16
Other sexual assault	7
IPVA	17
- Emotional	17
- Control [incl. via children and abuser’s family, ‘gaslighting’ i.e. being manipulated to question own memories/perceptions/beliefs]	17
- Physical	11
- Sexual	10
- Financial	6
	4
IPVA only	3
Both	15

Survey information on socioeconomic classification was not taken

^*^GCSE are school exams taken at 16 years old in UK and NVQ/BTEC are vocational qualifications

^**^ A levels are exams taken at 18 years old in UK

**Table 2:** Services Used

Services Accessed	*n *= 18 (16 women, 2 transgender) 19–25 years		
	Set I = 11 through third sector frontline agencies [more victimisation and socioeconomically disadvantage]		
	Set II = 8 through university events, online groups [less victimisation, more advantaged, more education, no children]		
	I & II	Set I	Set II
**-** Primary care [GP]	17		
**-** Secondary/crisis care	7		
Counselling [any]	16		
**-** School	10	5	5
**-** CAMHS*	10	6	4
**-** NHS [post 18]	9	5	4
**-** Private	3	0	3
**-** University	2	0	2
Police	12	10	2
Justice system	5	5	0
Social Services	8	6	2
Foster care	4	4	0
Third Sector support agencies	14	11	3
Safe house/hostel	6	6	0

**Table 3:** Role of Services in UK

Service or Role	Description of Service or Role
General Practitioner (GP)	Primary care—free usual first point of contact with NHS. Can refer on to specialists (clinical or community)
Maternity	Pre/post-natal care of women and babies is free within NHS
	Contact, advice and tests from health care professionals (HCPs)
	Several contacts in pregnancy and after birth
	Referred by GP or local hospital maternity unit
	Most women in UK have contact with the services
Third Sector Organisations	Non-profit, non-governmental organisations
	Raise funds to invest in social objectives
	Eg. charities that support YP with mental health problems or victims of DV or IPVA
	Services not always specialised in IPVA
	Referral pathways to organisations are varied
Counselling	Counselling services can be accessed free in UK
	Through health services, schools, colleges, universities or non-profit specialist organisations
	Individuals can refer themselves to private, fee-paying counselling
Support Worker	Professional who looks after the well-being of vulnerable people
	Can work for health, government or third sector organisations
	Includes a wide variety of roles—help people to live their lives as independently as possible
	Support to help individual reach their potential with practical and emotional support
	People are referred through the support organisation based on need

All (*n* = 18) of the YP had experienced at least one abusive intimate relationship. None were currently in an abusive relationship. Most (*n* = 15/18) had also experienced DVA in their family of origin. DVA always occurred alongside other negative life events. All participants had experienced at least one type of victimization including sexual abuse by adults or peers, emotional and physical abuse by parents, neglect and severe bullying by peers. More than half of the participants were recruited through frontline services (*n* = 11); these tended to be the more disadvantaged young women who had a long and complex history of negative life events, who had attempted help-seeking over the life course and prior to their IPVA. Participants recruited through university events, services or through online groups tended to come from more socio-economically advantaged backgrounds with less severe overall victimisation. For example, there were fewer instances of severe background DVA in the family of origin, including familial physical, sexual and emotional abuse, and in two cases, no DVA was reported. Nevertheless, there were still several cases within this group who had experienced multi-victimisation.

The current article’s themes were developed from the codes that clustered around: accounts of what had happened during intimate abusive relationships; which services were accessed and participants experiences of them; reflections following the end of their relationship(s) about what would have supported them, and what would help other young people to avoid abusive relationships in the future.

Institutional support and services accessed by YP were: 1) School 2) Health services including primary care physicians and maternity services; 3) Third sector (non-governmental or charitable) services; 4) Police. YP also engaged with two professional groups that could be accessed across several of the services: 5) Counselling; and 6) Support workers. When reflecting on their life histories and use of services, YP commonly mentioned what did/did not help, their ideas about what would help others, and turning points in their understanding about their IPVA.

The following sections report on participants’ experiences and outlines their perspectives on IPVA prevention and interventions needed.

## Institutional Support and Services Used

### School

England now has statutory relationship and sex education provision (Dept of Education, 2019). However, this was not the case for YP in the current study who were talking about their experiences prior to this change. Participants’ experience of schoolteachers and school counselling responses to their DVA-related help-seeking (i.e. related to either DVA within family of origin or IPVA within own intimate partner relationships) or acting out of distress has been reported elsewhere (Barnes et al., 2022) and were, for the most part, negative. The current section is concerned with the participant accounts of what they felt was missing, or might have helped, in terms of education about IPVA.

As reported in other literature (Davies, 2019), most participants in the current study were not aware that their partner was being abusive until late into the relationship or after it had ended. There was a disconnect between what they thought abuse was and their own experiences. Lack of information as to what constituted abuse in school relationship and sex education provision meant they did not have the tools or understanding to deal with the IPVA. Consequently, most participants generally assumed that sexual assault could not happen in a relationship and that abuse was to do with physical aggression; and lacked understanding about coercive control:

*I think that I really, really, struggled to understand or pinpoint controlling behaviour and mental abuse*. (Nive 24)

Personal experience led participants to identify elements that they would have found useful in relationship education, for example if they had been told *‘they’re meant to make you feel happy’* (Nancy 19) or it had been explained how early DVA within your family of origin can increase your vulnerability to further victimisation, especially IPVA:

*this happens, abuse, you do find your way to being a target for more abuse*. (Nina 23)

It was pointed out by a transgender participant that schools need to acknowledge LGBTQ + experiences and more effectively support them due to their vulnerability and isolation:

*[…] kids that don’t have that group of queer friends…they’re more likely to settle for whoever shows up…so they end up falling into that pattern [of abuse] and then they think they can’t get out*. (Xan 19)

Another participant explicitly noted how gender inequalities enabled abuse to occur in her own relationship and asked for schools to address this explicitly:

*A good dose of feminism in this period of my life would have been brilliant, because all I saw was I had very sexist teachers. I’d grown up in a very sexist environment, and I was very self-hating as a woman*. (Eleanor 19)

### Health Services

#### Primary Care Physicians (General Practitioners (GP) in UK)

Seeing a GP can be the first step in talking about symptoms related to the impact of early DVA within your family of origin or IPVA within one’s own intimate partner relationship, as well as accessing specialist support services via GP referral. However, approaching GPs for help was often seen as difficult, especially for more disadvantaged and multi-victimised participants. They rarely felt confident speaking with a professional with perceived authority about their problems, especially if they had previously experienced maltreatment *from* an adult with perceived authority.

The current structure of primary care often made it difficult to see the same GP and build a trusting relationship that could lead to disclosure and referral to specialist services. Having to repeat difficult conversations to different GPs for the most vulnerable could be daunting:

*To say there are no [IPVA] services out there is wrong, there are… If it wasn't for the fact that I went to my doctor's- but going to the doctors can be a scary thing. Saying ‘I feel depressed,’ especially with me, when I don't see the same doctor, it's really hard to build those relationships with doctors …having to explain the same thing over again, you get to a point where you just don't bother. So, I think these services need to make themselves a lot more approachable*. (Chloe 23)

The need for continuity of health care for IPVA survivors has been highlighted in previous studies (Pitt, 2018). Being ‘approachable’ facilitates consistency of health care and supports the development of a trusting relationship with health professionals. Approachability is essential given the unique role of GPs as gatekeepers to specialist services needed for IPVA recovery.

As with earlier research (Evans & Feder, 2016), specialist IPVA support only came via a few GPs, despite the fact that most participants in our study visited their GP due to mental health problems associated with either familial DVA or IPVA within intimate relationships. However, there were signs that some GPs did refer some of the young women in this study to specialist services, which were able to support participants in their recovery from IPVA.

*I went to my doctor, I felt really depressed because of this [online grooming/financial] scam. They referred me saying ‘Oh, have you ever heard of [service for vulnerable YP]? I said ‘No.’ It was me and my dad that went. It was to do with debt, but then I started to talk about [abusive partner], …because that was making me depressed as well. They were more interested in that than my debt in the nicest way*. (Bec 22)

Through support from her youth worker, Bec was able to move away from her abusive partner. Effective IPVA identification through primary care was rare for participants. However, once disclosure or identification had been made, there appeared to be a clear and effective pathway to specialist support.

#### Pregnancy/Maternity Services

Whilst pregnancy and motherhood can increase the risk of IPVA among all age-groups (Wood & Barter, 2015), for some of our participants it was also an important driver in starting the process of disengaging from an abusive partner. Many young women identified it as a turning point; they wanted a better life for their children than they had experienced and recognised the damage growing up around DVA can have on children:

*I was about two months pregnant, I just knew that Dan was never going to change. He wasn’t going to get a job, wasn’t going to sort himself out, wasn’t going to be responsible, because he just isn’t… I don’t want my son to have the life I did. It’s as simple as that*. (Chloe 23)

Young mothers are in constant contact with health professionals throughout their pregnancy, labour and postnatally, with the associated opportunities for service intervention. For example, Gemma spoke about how a healthcare professional interpreted her partner’s behaviour as controlling, enabling her to realise his behaviour constituted abuse:

*Literally within the first twelve hours of her being born, I held her once. He [ex-partner] had her in his arms for twelve hours straight. Which was all noted down in my maternity notes…it was only when my midwife put two and two together and said ‘that’s not love, that’s possession.’ I was like actually, yes, it is*. (Gemma 21)

Nevertheless, other chances for prenatal and postnatal health care professionals to intervene were potentially missed, and questions/discussions during pregnancy was rarely mentioned by other YP. This likely reflects the challenges of screening for IPVA in healthcare settings including lack of training, time, privacy, guidelines, policies, appropriate support from the employer (Kirk & Bezzant, 2020) and lack of robust evidence (O’Doherty et al., 2014).

### Third Sector Organisations

Local third sector organisations had helped the YP in several ways, including providing safe housing, help with unemployment benefits and IPVA-specific support. In participants’ experience, the Freedom programme n.d. delivered by the third sector was named as the most useful and supportive source of education and support. Freedom (www.freedomprogramme.co.uk) is a group programme for women over 18, who may or may not have separated from their abusive partner, to help name and recognise negative behaviours in order to understand IPVA. Participants appreciated how the different components of the course had helped them emotionally and practically. They gained support from other women in similar situations who did not judge them and eased feelings of isolation and loneliness (Barnes et al., 2022). Information provided by facilitators, on how abusive relationships work, gave them the knowledge to identify abuse in their own experiences:

*They [Freedom facilitators] are really, really good, just to show you all the different traits of a man…, you've got loads… the head worker, controller, persuader, the liar… and how it affects the [your] child*. (Soraya 22)

The programme was seen as a positive model to be used throughout school years to educate about IPVA effectively:

*School should do more awareness, but maybe when you're 12 and then all the way through. A bit like the Freedom [programme]. It should be like that*. (Nina 23)

Our participants had accessed the programme through special agencies and represented the most disadvantaged and multi-victimised group, while none of the YP recruited to the study through other avenues (e.g. university events) had accessed Freedom or specialist services. This highlights a possible weakness in the referral pathway due to lack of knowledge about the programme outside of third sector organisations.

Most participants wanted more information on available services, earlier in the referral process, and with more outreach:

*Just letting people know ‘this is the service we have [about IPVA]’… It's quite a daunting thing to do… Or a text system, where someone can just send you a text saying 'Need some guidance.'* (Chloe 23)

Whilst only one of the participants was a transgender woman, it was clear that she did not have information on specialist services where she would have felt safe attending:

*I think being transgender there is a lot of domestic violence and support services targeted for women and I guess, as a trans woman, that makes me feel like it’s not necessarily somewhere I can go because I don’t know if they’re going to accept me.* (Lara 25)

They felt that knowledge of more specific support, such as that provided by lovingme.uk ( Online support for trans and non binary domestic abuse survivors (lovingme.uk) for IPVA in the trans and non-binary community, could have been beneficial.

### Police

Police were often involved in participants’ IPVA help-seeking over an extended period of time. Typically, participants mentioned several police interventions having occurred before the relationship had ended. Police contact was felt to be a turning point, not least as a means of referral to formal support. A change in UK policing in the last decade to investigate IPVA (and DVA more broadly) more proactively—with a view to building an evidence-led case and not relying on the support of the victim (College of Policing, 2022) – was appreciated, and helped some participants begin the process of ending the relationship:

*I think the police were brilliant when they took it out of my hands. … now the things that they do as well when they listen to the phone calls, phone calls are constantly recorded. Before, if you didn’t let them in and you’d say ‘Everything’s fine’ that would be about it. They walk in now and need to check your whole environment before they leave*. (Nina 23)

However, not all accounts of police interventions were positive. One participant spoke about how the police had removed her ex-partner after she was raped, then left her on her own afterwards. Involvement of the police only rarely translated into any sort of custodial sentence for the abusive partner. Some participants reported long delays in receiving information about the prosecution of their abuser and the judicial process.

### Counselling Services

**Fig. 1:**
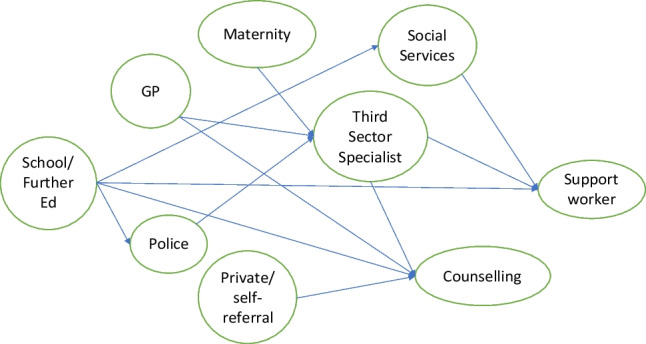
Referral Routes Described by YP experiencing IPVA

Counselling services could be accessed in a range of ways (Fig. 1). Therapeutic relationships were described positively by some participants, specifically if the therapy and therapist addressed the needs of the participant and helped build resilience:

*It really helped quite a lot…my therapist was trans, he really understood the trauma and abusive relationships and stuff*. (Lara 25)

However, Lara had to pay privately to find a suitable therapist. For most other participants, paying for counselling was not a possibility and waiting lists for free health service therapies were often long, leaving vulnerable people unsupported:

*I wanted to do psycho-educational art therapy, there was a very long waiting list for that…she was absolutely lovely, we covered a lot and we did a lot in that time*. (Ellie 25)

Many participants wanted counselling to help with the impact of IPVA, although most were ambiguous about receiving therapy. Targeted counselling for specific needs (e.g. for peri- or postnatal depression) helped. There were, however, adverse effects on participants’ lives (including abuse escalation) when inappropriate counselling for IPVA occurred:

*they put me onto a perinatal team at the Hospital… she [counsellor] was amazing. …through my pregnancy, with her, it helped a lot. [But] Because she used to try and do a lot of relationship counselling [with me], as well, so that she could maybe help with [abusive partner]. Which actually made it [the abuse] worse, because he didn’t want to hear what I was saying*. (Aaliyah 25)

The unintended consequences of what was viewed as an inappropriate intervention led to some withdrawing from therapy:

*I was seeing a therapist at the time for my anxiety, and she, I would say rather inappropriately, handed me a sheet about gaslighting in the middle of one of our sessions, which just panicked me, and I did stop seeing her after that. I still don’t think that was the right way to do it, and I left*. (Nancy 19)

Gaslighting is a commonly used term for a form of manipulation that makes someone question their own reality and if used in abusive relationships.

Another of the most vulnerable participants described having her needs and wishes over-ridden by her therapist. Due to Jackie’s learning disability her therapist assumed Jackie did not know what was in her best interest, thus recreating previously experienced uneven power dynamics:

*Actually, I wanted to finish counselling for ages. …I was going over the same subjects over and over again*. (Jackie 25)

Participants also wanted a more unified approach to counselling services, reporting frustrations with child and adolescent mental health services (CAMHS) ending at 18. They argued that support for the transition into adulthood was particularly needed to continue to build resilience:

*After you turn 18, they just kind of go ‘You're an adult now. Cheerio’ but it was in the middle of when I was going through everything, so it was a bit… I wouldn't say that triggered it [‘breakdown’], because I was already going through it a year before, but I think it probably wouldn't have lasted as long*. (Chloe 23)

Participants leaving home for university also left behind consistent health care for mental health problems and described how they stopped taking medications or receiving counselling. For Lily (22), it was part of the reason she spiralled into an abusive relationship with a drug dealer as ‘*it was very, very, easy for me to just drop off the map’.*

The findings highlight the importance of referral to appropriate and consistent counselling services, as well as appropriate counsellors within a service, that are trauma-informed and IPVA trained.

Trauma-informed practice is an approach to health and care interventions which is grounded in the understanding that trauma exposure can impact an individual's neurological, biological, psychological and social development (GOV.UK, 2022).

### Support Workers

Over half of the YP had had contact with a support worker and it was notable that all of them had very positive accounts of their experiences. Participants appreciated the equality and quality of their relationships with someone they considered to be a substitution or replacement for their own family.

For those who struggled with isolation and loneliness from early maltreatment and IPVA (Barnes et al., 2022), the modelling of a ‘good’ relationship with their support worker aided improvements in their health, wellbeing, and the development of their sense of agency:

*Being with [support worker], it’s really boosted my mood…I think it’s because I see her every week. She always says to me ‘Do you want me to wear my badge? I said ‘Oh, I don’t want you to wear it.’ We just go out for a coffee, do shopping. We do everything together, like your sister would…I couldn’t get better help from her. She is absolutely awesome*. (Bec 23)

Aside from consistency of support, Eleanor’s description of her support worker highlighted a key theme running through many participants’ accounts: helpful support included not judging the perpetrator, but supporting the victim:

*I saw him [disability support worker] weekly throughout the time I was [at university]… he was brilliant… It was just ‘What is best for her?’ Like having a dad at university. I didn’t need someone to condemn [partner], I needed someone to support me****.**** That is what he provided*. (Eleanor 19)

The care and quality of this support, and, importantly, working in partnership with the service user, enabled isolated YP experiencing IPVA to work their way through and end an abusive relationship. It also helped them to deal with the after-effects of the abuse. Support workers were most often mentioned by the more disadvantaged participants who had (usually) accessed them through third-sector support organisations.

## Discussion

The current paper aims to understand YP’s experiences of institutional support, services, or professional groups for IPVA; what helped and what would help in terms of prevention, intervention, and support for YP in abusive relationships.

A range of factors impacted on participants’ experiences of service provision: how appropriate the intervention was; being listened to; the consistency and quality of provision; and equality within the professional relationship. This held true across participants from a variety of backgrounds. The interventions discussed did not always specifically target IPVA, as YP often accessed services for other reasons, most often for mental health problems.

When reflecting on the most effective way to help YP with abusive partners, participants primarily concentrated on education and prevention. Participants, particularly those who had taken part in a specialist programme teaching them how to ‘see’ abuse, felt that information and opportunities for discussion on what abusive behaviour looks like, should be regularly provided in all schools. This supports UK findings where YP wanted earlier, interactive, relevant and accessible relationship education and information to help them to identify abusive behaviour (Griffiths, 2019; Heywood et al., 2019; Farrelly et al., 2022). As with recent work co-produced with YP and SafeLives Report (2020), participants wanted information to be able to make up their own minds without feeling judged.

Statutory guidelines in England on relationships education (Department of Education, 2019) state that schools have a duty to offer teaching on what is acceptable and unacceptable behaviour in relationships, and address IPVA and controlling behaviour ‘sensitively and clearly’. However, there is no specific guidance for best practice as to how this should be delivered, by whom, and what resources are available. The YP’s accounts in the current study indicate that these factors matter for YP, and such guidance is needed.

As found in previous studies, GPs are in a position to help YP receive appropriate IPVA support (Evans & Feder, 2016). Our findings showed that IPVA disclosure to a perceived authority figure can be difficult, with the perceived power differential within the GP encounter being a strong barrier for vulnerable YP to overcome. Participants stressed the desire for conversations with GPs to be made easier with the potential positive benefits of alleviating IPVA-related health problems and referral to specialist services.

Consistent with previous research, pregnancy and birth increased the risk of IPVA but, paradoxically, it was also a turning point for participants in leaving an abusive relationship (Wood & Barter, 2015; Wood et al., 2011). Participants also identified clear opportunities for health care professionals to intervene. However, anxieties about potential disclosure from patients and a lack of IPVA knowledge among health care professionals (Kirk & Bezzant, 2020) are exacerbated for midwifery staff, as partners are often present with the mother, and professionals can fear compounding the situation (Eustace et al., 2016). Structural change is needed to address the barriers in practice, including more time in consultations, and to improve privacy, training, policies, and referral protocols (Hudspeth et al., 2022).

The police are one of the main referrers to specialist services for YP experiencing IPVA. Frontline officers were often perceived as helpful when attending emergency calls. These findings differ from earlier literature where few YP felt police involvement had not been helpful in preventing further abuse, and police involvement often led to the victim dropping the charges (MacNab, 2010). The difference found probably is a reflection of the changes in police practice when attending domestic violence calls. However, similar to previous studies, participants also felt that follow-up care and information about the justice process was lacking (MacNab, 2010).

Most participants appreciated aspects of their therapeutic experience. However, many had also found counselling to be inappropriate or inadequate in addressing their specific needs. As found in other studies (Trevillion et al., 2012; Hameed et al., 2020), needs will not necessarily be met when psychological counselling is not trauma-informed. We found that YP were unlikely to present IPVA as their main reason for accessing counselling. As previously reported (Ferrari et al., 2014), clinicians need to be aware that patients presenting with mental health conditions or symptoms of depression or anxiety may also be experiencing IPVA or have a history of familial DVA.

YP highly valued the intervention/support received from support workers; echoing findings reported by older women accessing DVA or IPVA services (Stanley et al., 2021). The equal nature of the relationship with the support worker enhanced their wellbeing and stood in contrast to the power differential reported in with other professionals. For YP who have experienced previous relationships where power has been abused, it is likely that equity played a pivotal role in supporting and building resilience. This finding supports the small, but growing body of evidence on the support needs of young people who have experienced IPVA. In particular, and as with older adolescents in Källström & Thunberg’s study (2019), the current findings emphasise the importance of reducing the power inequality present in counselling relationships. These positive accounts of relationships with support workers can feed into trauma-informed training of other professionals (i.e. GPs) who encounter IPVA survivors. Any support approach should be sensitive to the previous adverse experiences of young people and should take care not to re-traumatise the individual.

Due to ethical considerations, all participants already had access to some sort of support. This meant that those recruited through frontline support organisations were from more disadvantaged backgrounds and multi-victimised than the general population. However, we also recruited through university groups and online support groups and included a range of backgrounds and experiences.

It seems that an equitable, person-centred and consistent relationship with services was desired by all participants to help support them during or after IPVA. It was clear that the most disadvantaged YP needed more of this support and for longer, as they often had more severe familial DVA and less stability and resources in their lives. They were also more likely to have children. As recommended by Korkmaz (2021), researchers, practitioners and policymakers need to be sensitive to how different societal positions interact and affect youth victimisation and young people’s ability to end abusive relationships.

Our findings illustrate the clear desire from YP for more detailed information and appropriate support to make their own decisions. Crucially, trust can be built with services and professionals when participants see that support is centred on their needs, at their pace, and in an equitable relationship (Stanley et al., 2021). Frontline professionals across sectors need trauma-informed training and a clear understanding of referral pathways to ensure that YP receive appropriate information and support. Sharing intelligence about barriers and facilitators between services about responding to survivors’ needs can inform the approach of services and professionals.

## Strengths and Limitations

The sensitive nature of the research meant that recruiting could only take place through organisations or via groups where participants could access support after their interview if necessary. As a result, we recruited participants who were more likely to have been ‘multi-victimised’ and had experience of some form of counselling. Therefore, our findings and conclusions can inform services mostly about how those who have been severely victimised might be better supported.

Sometimes, the LHC could be a useful distraction for participants who felt initially uncomfortable in the face-to-face interview setting; i.e. it gave them something to do or look at. The researcher found it useful to be able to check the LHC for blank spaces and ask ‘what was going on here?’ and it could elicit recall in a way that ‘normal’ questions had not reached. Finally, at the end of the interview, most participants found seeing the finished LHC—and sometimes seeing patterns within their history – as satisfying and/or enlightening.

The original aim of the study was to recruit and interview young female and male adults. We had difficulty engaging support organisations specifically for men to help us recruit. Nevertheless, we recruited YP from a variety of backgrounds and, through the use of Life History Calendars, addressed the recall bias that is present when interviewing participants about past events.

## Conclusions

These accounts of YPs experiences of universal and specialist services, has contributed to the field by identifying factors which facilitated or hindered their journey to recovery from familial DVA or IPVA.

There are several points of prevention and intervention that YP feel can increase IPVA awareness, help young people experiencing abuse, and alleviate the adverse impact on their health and wellbeing. GP and maternity services and counselling services are uniquely placed to effectively address the experiences of IPVA survivors; it is important these services being well trained in trauma-informed care (including the relevance of power dynamics) and referral pathways. Third sector specialist services and programmes for YP experiencing IPVA are helpful and valued in the process of building knowledge and strength to leave an abusive partner. Support workers are well placed to help this process and, as such, more consistent funding for these programmes, organisations and roles are needed. Appraisal of the content and consistency of approaches to relationship education – especially abusive and controlling aspects of relationships—across schools, including the engagement of students from all backgrounds, sexualities and identities is also needed.

## Data Availability

The qualitative datasets generated and analysed during the current study are not publicly available due to the highly sensitive nature of the research and the need for strict anonymity and confidentiality for the participants involved. University of Bristol Ethics Committee Approval (ref: 83722) 2019 for the YARAH Study. All methods were carried out in accordance with relevant guidelines and regulations. Informed, written consent was obtained from all subjects.
